# CARD15/NOD2 Is Required for Peyer's Patches Homeostasis in Mice

**DOI:** 10.1371/journal.pone.0000523

**Published:** 2007-06-13

**Authors:** Frédérick Barreau, Ulrich Meinzer, Fabrice Chareyre, Dominique Berrebi, Michiko Niwa-Kawakita, Monique Dussaillant, Benoit Foligne, Vincent Ollendorff, Martine Heyman, Stéphane Bonacorsi, Thecla Lesuffleur, Ghislaine Sterkers, Marco Giovannini, Jean-Pierre Hugot

**Affiliations:** 1 U843, INSERM, Paris, France; 2 UMR-S843, Université Paris Diderot, Paris, France; 3 Service de Gastroentérologie, Hôpital R. Debré, AP-HP, Paris, France; 4 Institut Universitaire d'Hématologie, Université Paris Diderot, Paris, France; 5 U674, INSERM, Paris, France; 6 EA3102, Université Paris Diderot, Paris, France; 7 Service d'Anatomie Pathologique, Institut Pasteur de Lille, Lille, France; 8 Laboratoire des Bactéries Lactiques et Immunité des Muqueuses, Institut Pasteur de Lille, Lille, France; 9 IMRN and UMR 1111 INRA, Faculté Saint-Jérôme, Université Paul Cézanne, Marseille, France; 10 U793, INSERM, Paris, France; 11 IFR94, Université Paris Descartes, Paris, France; 12 EA3105, Université Paris Diderot, Paris, France; 13 Service de Microbiologie, Hôpital R. Debré, AP-HP, Paris, France; 14 Service d'Immunologie, Hôpital R. Debré, AP-HP, Paris, France; Centre de Recherche Public-Santé, Luxembourg

## Abstract

**Background:**

CARD15/NOD2 mutations are associated with susceptibility to Crohn's Disease (CD) and Graft Versus Host Disease (GVHD). CD and GVHD are suspected to be related with the dysfunction of Peyer's patches (PP) and isolated lymphoid follicles (LFs). Using a new mouse model invalidated for *Card15/Nod2* (KO), we thus analysed the impact of the gene in these lymphoid formations together with the development of experimental colitis.

**Methodology/Principal Findings:**

At weeks 4, 12 and 52, the numbers of PPs and LFs were higher in KO mice while no difference was observed at birth. At weeks 4 and 12, the size and cellular composition of PPs were analysed by flow cytometry and immunohistochemistry. PPs of KO mice were larger with an increased proportion of M cells and CD4^+^ T-cells. KO mice were also characterised by higher concentrations of TNFα, IFNγ, IL12 and IL4 measured by ELISA. In contrast, little differences were found in the PP-free ileum and the spleen of KO mice. By Ussing chamber experiments, we found that this PP phenotype is associated with an increased of both paracellular permeability and yeast/bacterial translocation. Finally, KO mice were more susceptible to the colitis induced by TNBS.

**Conclusions:**

*Card15/Nod2* deficiency induces an abnormal development and function of the PPs characterised by an exaggerated immune response and an increased permeability. These observations provide a comprehensive link between the molecular defect and the Human CARD15/NOD2 associated disorders: CD and GVHD.

## Introduction

Caspase Recruitment Domain 15 (*CARD15*) also known as Nucleotide oligomerisation domain 2 (*NOD2*) has been associated with Crohn's Disease (CD) [1], [2] and graft versus host disease (GVHD) [3], [4]. *NOD2* belongs to a family of genes involved in innate immunity [5]. It can be activated by muropeptides which are components of the bacterial cell wall. When activated, NOD2 interacts with Rick/Rip2 which in turn activates the NF-kB pathway, resulting in the production of pro-inflammatory cytokines.

Half of CD patients have one or more *NOD2* mutations [6]. Some of the CD associated mutations were found unresponsive to muropeptides [5]. By consequence, CD is usually considered as an immune deficiency toward bacteria present in the gut lumen [7]. However, the exact mechanism by which *NOD2* mutations are able to induce CD lesions is still subject to debate [7]–[10].

*Holler* et al. reported that the three major mutations associated with CD (R702W, G908R and 1007fs) are also associated with severe acute GVHD and bone marrow transplantation (BMT) related mortality [3]. Mutations in both donor and recipient were found deleterious, suggesting a role of epithelial and circulating cells in disease mechanisms. Despite some differences in their conclusions, other groups recently confirmed the association between *NOD2* and BMT complications [4], [11].

CD is a chronic relapsing inflammatory bowel disease (IBD) with mucosal ulcerations of the digestive tract. CD lesions are characterised by a T helper (Th) 1 immune response and several authors have shown that they are related with gut associated lymphoid tissue (GALT), known as lymphoid follicles (LFs). LFs are mainly encountered in the colon where they are isolated and in small bowel where they are grouped forming Peyer's patches (PP) which are known to be pivotal sites for the host immune response and for the entry of enteropathogen bacteria. CD lesions are most often localized in colon and distal ileum, where the LFs are the most abundant [12]. *Fujimura* et al. found that aphtoïd ulcerations (which are often considered as the earliest CD lesions) are centred by LFs [13]. In addition to this spatial relationship, a temporal link between CD and PP development has also been suggested [14]. PPs develop from birth to 10–15 years of life and then undergo involution. The age-dependent incidence curve of CD is roughly parallel to the number of PP with a delay of about 10 years. Finally, ileal lesions are uncommon in young children and seniors where PP are rare [15], [16].

The role of GALT in GVHD has been suspected on both experimental models and clinical observations. Animal studies showed that death after BMT was prevented by gut decontamination and that prevention of mucosal damage also prevents lethal GVHD [17]. In clinical practice, gut decontamination reduces the frequency and severity of GVHD. Finally, *Murai* et al. recently showed that PP deficient mice are resistant to GVHD, arguing for a crucial role of PPs in GVHD, at least in models that do not use conditioning of the host prior to adoptive transfer of the allogeneic donor cells [18], [19].

Considering all these elements, we hypothesized that *Nod2* may play a role in the structure and function of the GALT. Consequently, we used a new mouse model deficient for *Card15/Nod2* i) to evaluate the involvement of Nod2 on the number and PPs size; ii) to assess whether *Nod2* modified cellular composition and cytokine expression of PPs; and iii) to determine whether *Nod2* may alter paracellular permeability and bacterial translocation of PPs in adult mice. Finally, we also examined if *Card15/Nod2* deficiency affects the colonic response to 2,4,6-trinitrobenzene sulphonic acid (TNBS), a classic experimental model of colitis in mouse.

## Results

**Figure 1:**
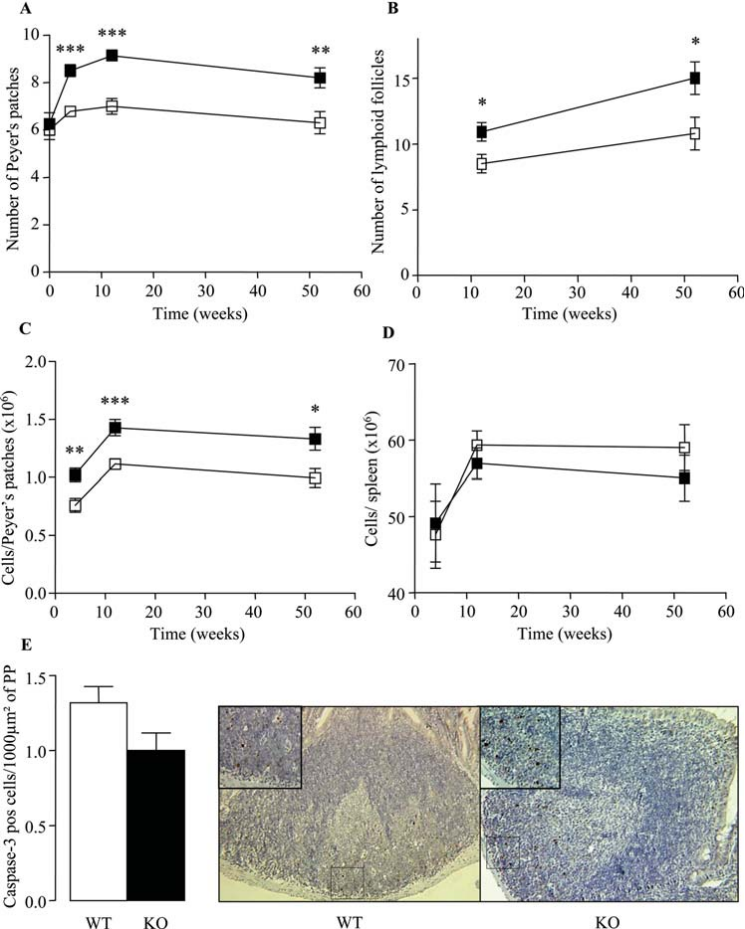
*Nod2* and postnatal development of gut associated lymphoid formations. (A) PP count on the whole intestines of KO (▪) and WT (□) mice at birth and at weeks 4, 12 and 52. (B) Number of isolated LFs identified on microscopic examination of the small intestine at weeks 12 and 52. (C and D) Number of cells per PP and spleen at weeks 4, 12 and 52). (E) Number of apoptotic cells identified by caspase-3 immunostaining at week 12. Data represent the means±SEM of 8 mice per group. *P<0.05; **P<0.01, ***P<0.001.

Body weight, intestinal length and intestinal weight were similar in KO and WT mice (supplementary information (SI) Table S1). Macroscopically, no inflammation was visible in KO mice according to Wallace and Ameho criteria (data not shown). KO mice exhibited an increased number of PP in comparison with WT mice at weeks 4, 12 and 52 after birth (Fig 1 *A*). At weeks 12 and 52, the number of isolated LFs per small intestine was also higher in KO mice than in WT mice (Fig 1 *B*). In contrast, the number of PPs at birth was similar between KO and WT mice (6.2±0.5 *vs.* 6.0±0.4; *P*>0.05) (Fig 1 *A*).

Macroscopically, the size of PP formations appeared to be larger in adult KO mice (data not shown). To quantify this difference, the three biggest PPs of each mouse were pooled and their cells were counted. At weeks 4, 12 and 52, KO mice exhibited a higher cell number per PP (fig 1 *C*). In contrast, cell counts were comparable in spleens of KO and WT mice (Fig 1 *D*).

Microscopic analyses of PP formations from KO mice revealed no gross abnormalities (Fig 1 *E*). Finally, as NOD2 modulates the NF-κappaB pathway and putatively the apoptosis, we investigated the number of apoptotic cells inside PPs. Immunohistochemistry experiments did not show differences between KO and WT mice for the number of caspase 3 positive cells (1.01±0.11 *vs.* 1.32±0.10; *P*>0.05) (Fig 1 *E*).

**Figure 2:**
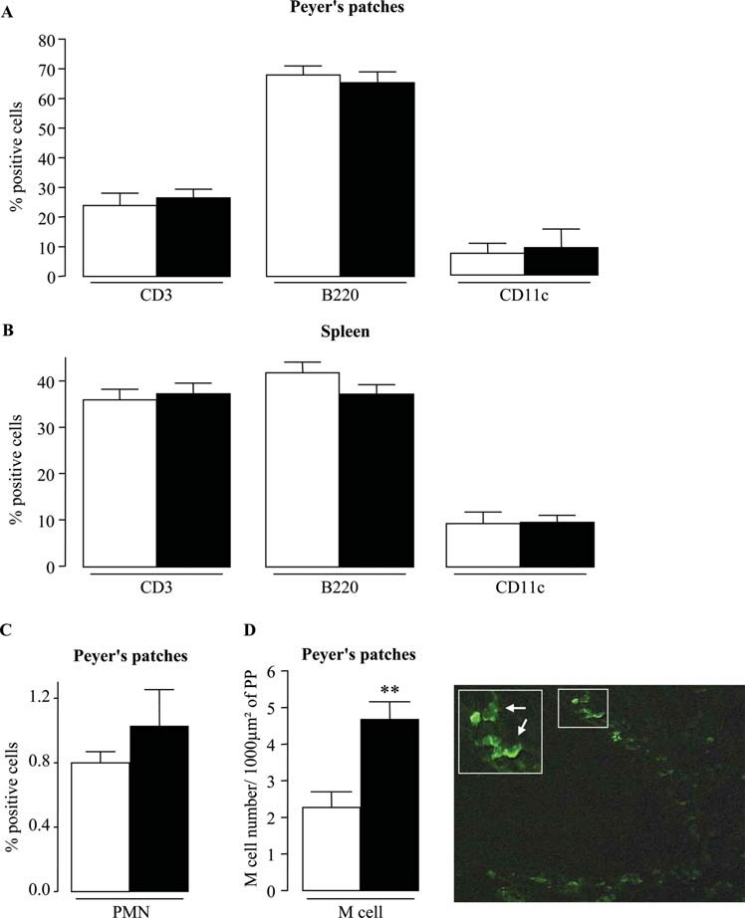
*Nod2* and Peyer's patch cellular composition. (A and B) Relative proportions of T-, B- and dendritic cells from PPs and spleens of KO (▪) and WT (□) mice at week 12. T-, B- and dendritic cells were investigated by flow cytometry using antibodies to CD3, B220 and CD11c. (C) Relative proportion of polymorphonuclear neutrophils was analyzed using Ly-6G antibody. (D) M cells number inside the follicle associated with epithelium was investigated by immuno-histochemistry. Arrows indicated the presence of M-cell inside the follicle associated with epithelium. Data represent the means±SEM of 8 mice per group. **P<0.01.

In order to better characterise the phenotype of the cells present in PPs, we performed flow cytometry experiments using the B220, CD3 and CD11c antibodies. At week 12, no differences were seen regarding the relative proportions of B220^+^ B-cells, CD3^+^ T-cells and CD11c^+^ dendritic cells between KO and WT mice either for PPs or spleens (Fig 2 *A*–*B*).

Because some cells present in a limited number may be functionally important, we also investigated the relative proportion of Ly-6G^+^ polymorphonuclear neutrophils (PMN) present in the PPs (Fig 2 *C*) and the number of M cells located inside the follicle associated epithelium (FAE) (Fig 2 *D*). No difference of PMN relative proportion was observed in PPs of KO and WT mice (Fig 2 *C*). At the opposite, M cell number was increased in the FAE of KO mice in comparison with WT mice (Fig 2 *D*).

**Figure 3:**
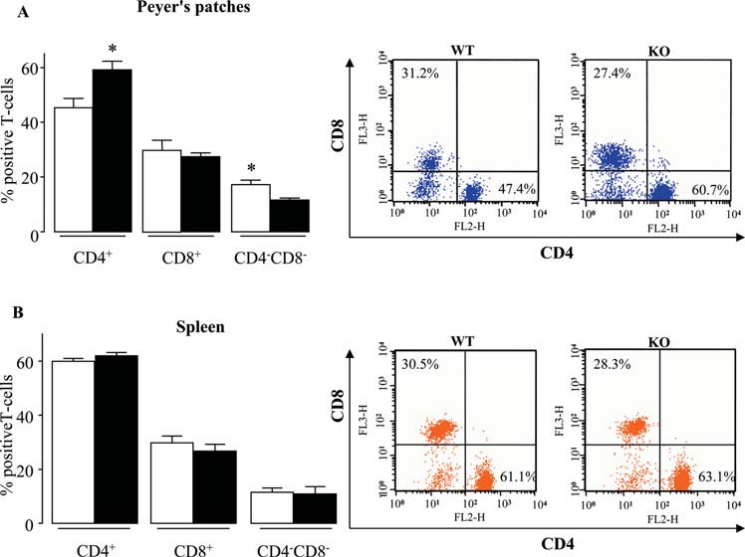
*Nod2* and T-cells subset in PP and spleen from mice at 12 weeks of age. Relative proportion of CD4^+^, CD8^+^ and CD4^−^CD8^−^ T-cells from PPs (A) and spleen (B) of KO (▪) and WT (□) mice. At week 12, CD3^+^ T-cells were stained with antibodies to CD3, CD4, and CD8. Data were gated for CD3^+^ T-cells. Data represent the means±SEM of 8 mice per group. *P<0.05.

As T-cells are known to play a pivotal role in CD, we further analyzed the CD3^+^ T cells. At week 12, KO mice exhibited an increase of CD4^+^ T-cell relative proportion within their PPs, whereas the proportion of CD8^+^ T-cells remained constant (Fig 3 *A*). As a mirror image, PPs from KO mice exhibited significantly fewer CD3^+^CD4^−^CD8^−^ T-cells (Fig 3 *A*). In contrast, no comparable differences were seen in the spleens (Fig 3 *B*). Similar data were obtained at week 4 (SI Fig S1 *A–B*).

Finally, we investigated the phenotype of CD4^+^ T-cells present in the PPs and spleens by examining the relative proportions of naive CD25^−^CD45Rb^+^, regulatory CD25^+^CD45Rb^−^ and memory CD25^−^CD45Rb^−^ CD4^+^ T-cells. KO and WT mice had a similar relative proportion of naive, regulatory and memory CD4^+^ T-cells in PPs (SI Fig S2 *A*) and spleen (SI Fig S2 *B*). Flow cytometry analyses also failed to reveal a difference between KO and WT mice when investigating the annexin V positive CD3^+^ and CD3^+^CD4^+^ T-cells extracted from PPs (SI Fig S2 *C*) suggesting that the excess of CD4^+^ T-cells observed in KO mice does not result from a defect of apoptosis.

**Figure 4:**
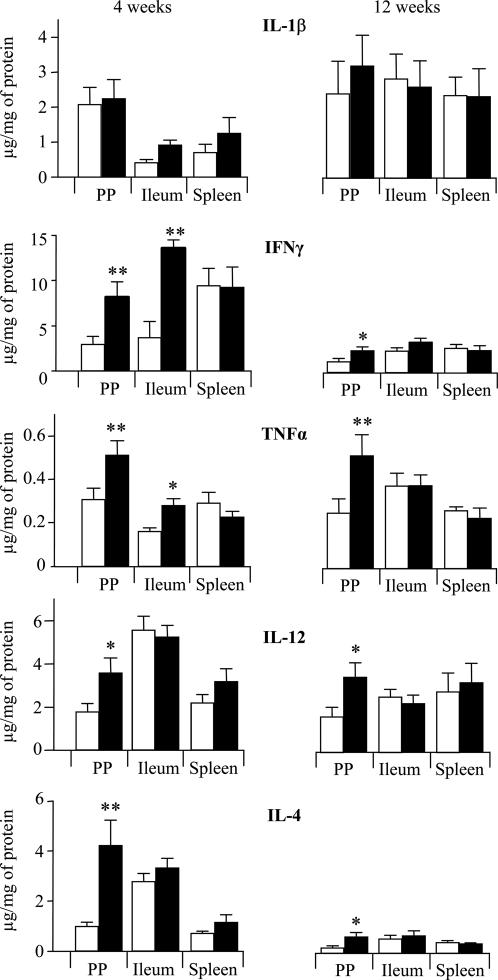
*Nod2* invalidation increases cytokine expression in PP. Expressions of Il-1β, IFNγ, TNFα, IL-12, IL-4 were determined by ELISA in PPs, Ileum and spleens from KO (▪) and WT (□) mice at weeks 4 (left panel) and 12 (right panel). Data represent the means±SEM of 8 mice per group. *P<0.05; **P<0.01.

Considering the differences of PP cell number and cellular composition, we further evaluated whether PPs from KO and WT mice might exhibit differences regarding their cytokine profile. Thus, the expression of IL-1β, IFNγ, TNFα , IL-12, and IL-4 in PPs, PP-free ileum and spleen were determined by ELISA, at weeks 4 and 12. Levels of IFNγ, TNFα, IL-12, and IL-4 were significantly increased in PPs of KO mice (Fig 4). In contrast, less marked differences were seen between KO and WT mice in PP-free ileum where TNFα and IFNγ were the only cytokines differentially expressed at week 4 but not in adult mice (Fig 4). Finally, no differences were observed in spleens from KO and WT mice (Fig 4).

**Figure 5:**
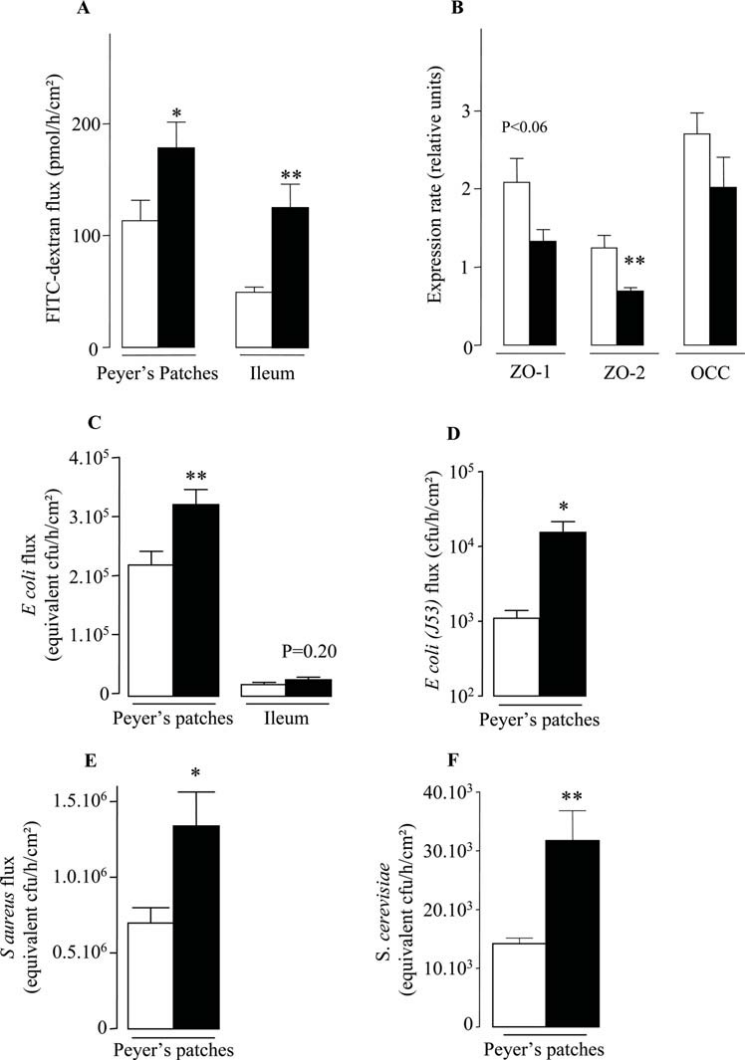
Paracellular permeability and bacterial translocation are increased in *Nod2* invalidated mice. Ussing-chamber and Real-time PCR experiments were performed on PP and PP-free ileum from WT (□) and KO (▪) at week 12. (A) Paracellular permeability was analysed by FITC-dextran flux from PP and PP-free ileum under basal condition. (B) mRNA expression levels of TJ proteins (ZO-1, ZO-2 and Occ) from PP were analysed by real-Time-PCR under basal condition. (C) Bacterial translocation of chemically killed fluorescent Escherichia Coli K-12. (D) Translocation of the viable non enteropathogen Escherichia Coli strain J53. (E) Translocation of a chemically killed fluorescent Staphylococcus Aureus. (F) Translocation of chemically killed fluorescein-conjugated *Saccharomyces cerevisiae*. Data represent the means±SEM of 8 mice per group. *P<0.05 and **P<0.01, significantly different from WT.

Cell composition and cytokine production may affect the function of PPs. We thus used Ussing chambers for determining the paracellular permeability through PP and PP-free ileum. PPs and PP-free ileum of KO mice exhibited a significant increase in paracellular permeability (Fig 5 *A*) while the electrical resistances were comparable (data not shown). In parallel to this change, mRNA expression levels of TJ proteins were affected in PP of KO mice. Indeed, expression of mRNAs encoding ZO-2 and ZO-1 were decreased by 45% and 36% (P<0.01 and P<0.06 respectively) (Fig 5 *B*). In contrast, Occludin expression was unchanged (Fig 5 *B*).

Because Nod2 is involved in innate immunity we hypothesised that *Card15/Nod2* deficiency may affect the gut microflora. We thus counted the numbers of bacteria classified as *Enterobacteriaceae, Pseudomonas, Staphylococcus, Streptococcus, Enterococcus or Lactobacillus* in the ileum of KO and WT mice. However, we did not observed differences between groups (SI Table S2).

Ussing chamber experiments were also performed to investigate the bacterial passage through PP. The passage of a chemically killed *Escherichia coli* (K-12) was higher in PP of KO mice (P<0.01) (Fig 5 *C*). This increased translocation was also observed using living non pathogenic *Escherichia coli* strain J53 (Fig 5 *D*). In contrast, translocation of *Escherichia coli* across free-PP ileum mucosa was very low and not significantly increased (*P*>0.05, fig 5 *C*). The translocation through PPs of a chemically killed *Staphylococcus Aureus* (Wood strain without proteinA) and *Saccharomyces cerevisiae* was also higher in KO than in WT mice (Fig 5 *E* and *F*)).

**Figure 6:**
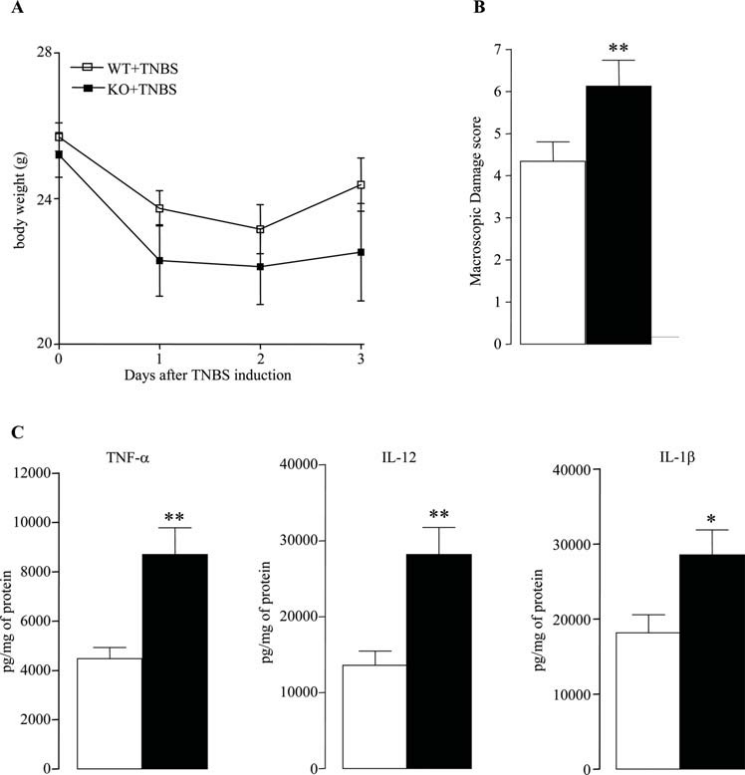
*Nod2* invalidation increased the suceptibility of TNBS induced colitis. (A) Body weight, (B) Macroscopic damage score, (C) cytokines levels were assessed in 12 week old mice. These parameters were determined three days after intracolonic administration of TNBS. Values are mean (SEM) (n = 8 in each group). *P<0.05 and **P<0.01 between WT and KO mice.

TNBS colitis is a well studied model of acute colitis in mice but no data are currently available in the literature on the effect of *Card15/Nod2* in this experimental model. Three days after intracolonic instillation of TNBS, the body weight loss was higher in KO than in WT mice but this difference did not reach significance (Fig 6 *A*). Nevertheless, the colonic mucosal damage score was higher in KO mice (Fig 6 *B*) as well as mucosal concentrations of IL-1β, TNF-α and IL-12 (Fig 6 *C*).

## Discussion

Since the discovery of an association between *CARD15/NOD2* mutations and both CD [1], [2] and GVH [3], [4], the pathophysiological functions of *CARD15/NOD2* involved in CD and GVHD development are poorly understood [9]. However, because an association between GALT dysfonctions and GVHD, as well as spatial and temporal links between CD lesions and PP have been suggested by several authors, we used a new model of *Card15/Nod2* deficient mouse, to explore the impact of *CARD15/NOD2* in PP development and function. We observed that adult KO mice exhibit an excess of PP and isolated LFs in the gut. PP from KO mice are characterized by an excess of M cells and CD4^+^ T-cells and a higher expression of Th1 and Th2 cytokines. These differences are associated with increased paracellular permeability and bacterial and yeast passage through PPs. Finally, we observed that *Card15/Nod2* deficient mice are more susceptible to TNBS induced colitis. As a whole, the here reported phenotype of the *Card15/Nod2* KO mouse is reminiscent to the observations made in the Human diseases associated with *CARD15/NOD2* mutations: CD and GVHD.

Our data demonstrate first that *Card15/Nod2* deficient mice have an elevated number of PPs and isolated LFs after birth. As intestinal weight and length are similar between KO and WT mice, this finding does not seem to be secondary to an intestinal overgrowth. In addition, PPs from deficient mice are larger, as indicated by the macroscopic examination of the intestines and by the count of PP cells. As a result, *Card15/Nod2* modulates the development of the GALT and *Card15/Nod2* deficiency is characterised by an overgrowth of the lymphoïd tissue present in the gut. This over-development of the lymphoïd tissue seems to be specific to the GI tract as non significant change were observed in a systemic immune organe like spleen. The lack of difference in PP number between WT and KO mice at birth indicates that *Card15/Nod2* plays its role during post natal development of the GALT. While lymphotoxin and IL-7 signalling are essential for the organogenesis of PP during the embryonic stage [20]–[22], it is widely believed that gut commensal bacteria are critical for the postnatal development of gut mucosal immune system, as demonstrated by studies on germ-free animals [23]. Such animals have an underdeveloped GALT and are resistant to experimental colitis and to severe GVHD [18]. Thereby, pattern recognition receptors, including TLRs and NOD molecules, can be seen as good candidates by which the resident flora stimulates the development of GALT. However, TLRs play only a limited role in PP development at early postnatal stage in mice [24]. At the opposite, our data suggest that *Card15/Nod2* plays a pivotal role in the postnatal development of GALT.

Because gut flora is important in PP development, it can be questioned if *Card15/Nod2* deficiency affects gut flora composition of the host. In order to answer this question, we counted the bacteria most represented in the ileum and able to cultivate in standard conditions. We failed to demonstrate differences between KO and WT mice suggesting that *Card15/Nod2* deficiency does not induce gross abnormalities of the gut microflora. However, more discrete alterations cannot be discarded and additional experiments using germ-free animals and/or molecular methods for bacterial detection are required to further explore the relationship between *Card15/Nod2* and the normal gut flora.

PPs have B-cell follicles and germinal centres surrounded by areas that contain predominantly T cells. The analysis of the PP from *Card15/Nod2* deficient mice failed to reveal gross abnormalities in terms of microachitecture, apoptosis or cell composition (at least for the three main cell lineages present in PP, namely B-cells, T-cells and dendritic cells). This observation is in accordance with the previous descriptions of other *Card15/Nod2* KO models of mice which failed to reveal gross intestinal abnormalities under basal conditions [7], [25]. Interestingly, the phenotype observed in the deficient mouse is reminiscent with the Human CD condition where large lymphoid aggregates with normal microarchitecture and cell composition have been reported [26].

LFs are covered by a specialised epithelium including M cells. These cells are able to transfer bacterial and food antigens from the gut lumen to antigen presenting cells [23]. They have thus a pivotal role in the function of PP. M cells were found more numerous in KO mice. In addition, PP of KO mice exhibited a higher proportion of CD4^+^ T-cells and a decreased percentage of CD3^+^CD8^−^CD4^−^ T-cells. As Nod2 may modulate the apoptosis, we have hypothesized that this increase of CD4^+^ T-cell number may result from an apoptosis defect in the lymphoid cells of KO mice. However, flow cytometry analyses revealed that the relative proportion of apoptotic CD4^+^ T-cells from PP was similar between KO and WT mice. As a result, our experiments do not support the opinion that *Card15/Nod2* deficiency is characterised by a general apoptosis defect in the lymphoid tissue of KO mice. Finally, the altered immune cell composition is concomitant with an increase of pro-inflammatory Th1 but also anti-inflammatory Th2 cytokine expression. Altogether, these results indicate that under basal condition, PPs of KO mice are characterised by an exaggerated immune response.

Cell composition and cytokine production may affect the function of PP. We thus used Ussing chambers for determining the paracellular permeability through PP and PP-free ileum. KO mice exhibited a significant increase of paracellular permeability. This result is in accordance with the altered intestinal permeability reported in CD patients and their healthy relatives, especially in case of *CARD15/NOD2* mutations [27]–[30]. Pro- and anti-inflammatory cytokines are known to modulate intestinal paracellular permeability. IFNγ, TNFα and IL-4, act on membrane receptors of epithelial cells to increase tight junction permeability [31]–[34]. For example, on T84 cells IFNγ decreased levels of ZO-1 and altered apical actin organisation, which leads to disorganisation of TJ and increased permeability [35]. Similarly, PP from KO mice exhibited a decrease of ZO-1 and ZO-2 mRNA expression in comparison with WT mice. Consequently, the excessive concentration of IFNγ observed in KO mice may down regulate the transcription of ZO-1 and ZO-2 mRNA expression and contribute to the increase paracellular permeability in KO mice. It is to note that this phenotype is reminiscent to the *CARD15/NOD2* associated Human disorders. Indeed, in CD patients, increased permeability has been reported to be mediated by TNFα and to precede the clinical relapse while GVHD has been treated by anti-TNF antibodies [36], [37].

Because of the changes in gut permeability, we finally studied the role of *Card15/Nod2* in bacterial translocation. Bacterial passage of *Staphylococcus aureus* and *Escherichia coli* and yeast ingress of *Saccharomyces cerevisiae* were higher through PP of KO mice. It is widely accepted that CD is related with an excessive bacterial translocation through the intestinal epithelium even if this hypothesis is not perfectly documented. The present data further support this opinion. In addition, this excess of yeast and bacteria translocation through PP of KO mice is in agreement with recent reports showing that mutated CD patients and their unaffected relatives develop more frequently antibodies to *Saccharomyces cerevisiae, Pseudomonas fluorescens*–related protein, *Escherichia coli* outer membrane porin C and CBir1 flagellin [38]. The excessive bacteria and yeast passage reported here may participate in the enhancement of adaptive immune responses to microbial antigens.

The increased number of M cells can contribute to the high translocation rate observed in KO mice. However, because M cell differentiation is inducible by microbial challenge [39], it may also be a consequence of bacterial translocations and additional experiments are required to further dissect this complex relationship. It is also possible to consider that *CARD15/NOD2* dysfunction facilitates bacterial entry through defective antibacterial peptide expression [7], impaired intracellular bactericidal capacity or reduced epithelial immune defence. Finally, bacterial translocation may also be secondary to primitive local cytokine changes. IFNγ is known to increase the epithelial adherence of selected species of enteric bacteria [40]. *Ferrier* et al. have shown that a chronic stress in mice drives an organ-specific cytokine expression pattern which in turn, alters the colonic mucosal barrier functions and favours bacterial translocation [31]. This effect is dependent on the presence of CD4^+^ T-cells and requires IFNγ production. It is thus possible that bacterial translocation is a result of the immune changes rather than its cause and additional experiments are now required to answer this question.

Finally, we have shown that *Card15/Nod2* invalidation exacerbates the severity of TNBS induced colitis, as evidenced by the increase in all parameters characterising colonic inflammation (damages scores and mucosal levels of IL-1β, TNFα and IL-12). This enhanced colitis-induced by TNBS might be explained by different mechanisms. Firstly, since microflora is required for TNBS-induced colonic mucosal damages and since *Card15/Nod2* signaling has been shown to inhibit the TLR2-driven activation of Th1 response [8], one can hypothesize that the Th1 inflammatory response mediated by TLR-2 is increased in *Card15/Nod2* deficient mice. Secondly, since GALT is reported to modulate the severity of TNBS-induced colitis [41], [42] it can be hypothesised that the TNBS induced colitis is exacerbated in *Card15/Nod2* KO mice because of GALT overdevelopment. Finally, an alternative explanation could be that the observed defect in terms of intestinal permeability and bacterial translocation makes the intestine more susceptible to TNBS.

Altogether, the present data demonstrate that *Card15/Nod2* is required to maintain the homeostatis of the PPs. Because *CARD15/NOD2* is a well demonstrated etiological factor for CD/GVHD, this conclusion is of particular importance for the understanding of disease mechanisms. Indeed, it further supports the opinion that the defect involved in the development of the gut lesions is related with PP and LF function. The GALT dysfunction observed in KO mice is associated with an excessive gut immune response and an increased bacterial translocation. These findings are consistent with our knowledge on the Human diseases associated with *CARD15/NOD2* mutations namely GVHD and CD and for which similar observations have been reported. As a result, the phenotype of the *Card15/Nod2* KO mice can be seen as an attenuated model of CD/GVHD. It is to note that the absence of a full phenotype is not unexpected considering the multifactorial nature of the Human diseases where exposure to several additional unknown genetic and environmental risk factors is required for disease expression. At the opposite, our work indicates that the *Card15/Nod2* KO mouse is a relevant model for investigating these other risk factors associated with CD and GVHD.

## Material and Methods

### Animals

**Figure 7:**
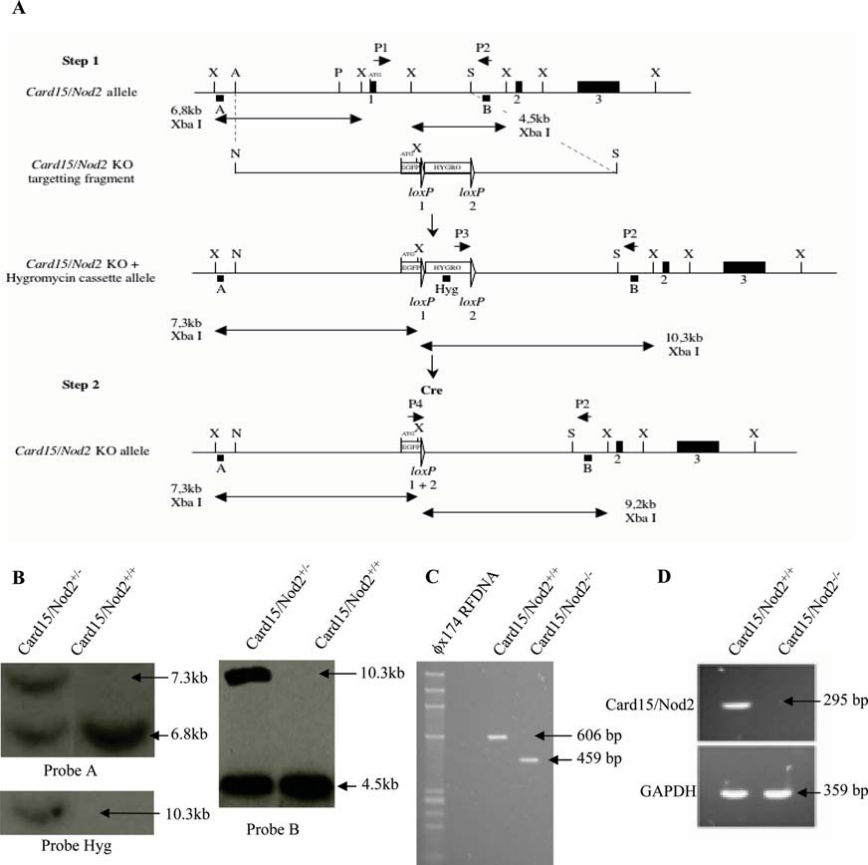
Targeting disruption of the murine *Nod2* gene by homologous recombination. (A) Generation of the Card15/Nod2 KO allele: targeting strategy. The restriction maps of the *Card15/Nod2*+allele (5′ portion), the *Card15/Nod2* KO targeting fragment, and the modified *Card15/Nod2* allele after homologous recombination and Cre-mediated recombination of an Hygromycin selection cassette are shown. Exons 1, 2, and 3 (black boxes) and restriction sites used for cloning and screening (X) XbaI, (A) AgeI, (P) PmlI, (S) SalI, (N) NotI are indicated. The *Card15/Nod2* KO targeting fragment comprises the EGFP gene (Clontech) in frame with *Card15/Nod2* ATG and the floxed PGKHygromycin (Clontech) selection cassette, introduced between the AgeI site downstream of *Card15/Nod2* exon 1 and the SalI site upstream of exon 1 in the *Card15/Nod2* orientation. All loxP sites are represented by open triangles. Recombination of loxP1 and loxP2 results in the loxP1+2 site. In the first step of the strategy, the *Card15/Nod2* locus was targeted with the *Card15/Nod2* KO targeting fragment. In the second step, the PGKHygromycin selection cassette was removed by Cre recombinase. The double-headed arrows indicate the DNA fragments resulting from digestions with different enzymes expected to hybridize with probes A, B or Hyg. Also depicted are combinations of PCR primers P1-4 that detect the different *Card15/Nod2* alleles. (B) Southern blot analysis of restricted DNA resulting from digestion with XbaI.Hybridation with Probe A and B show complete integration of the targetting fragment after homologous recombination. Probe Hyg show the differents integrations of targetting fragments after homologous recombination. (C) Genotyping of Nod2-deficient mice by PCR. Genomic DNA from mice was amplified by PCR to detect the disrupted sequence (PCR product of 459 bp). (D) Expression of Nod2 mRNA in spleen. RT-PCR was performed on purified mRNA from the spleen of WT and KO mice. As expected, no signal was observed in KO mice. GAPDH expression was used as positive control of expression.

*Card15/Nod2* was disrupted in mice by replacing the first coding exon carrying the majority of the sequence encoding the CARD domains including the start codon with a EGFP cassette. (Fig. 7 A). In the first step of the strategy, the *Card15/Nod2* locus was targeted with the *Card15/Nod2* KO targeting fragment. In the second step, the PGKHygromycin selection cassette was removed by Cre recombinase. Targeted disruption was determined by Southern blot and long-range PCR analysis (Fig. 7 *B*–*C*). Genomic DNA from mice was amplified by PCR using the primers: 5-GTCATTTCCTGACCTCTGACC-3 and 5-AACCGCATTATTCCATGGGGC-3 to detect WT DNA and primers 5-AACCGCATTATTCCATGGGGC-3 and 5-GCCTGCTCTTTACTGAAGGCTC-3 to detect the disrupted sequence. The loss of mRNA *Nod2* expression was demonstrated in splenocytes by RT-PCR (Fig. 7 *D*) using the following primers: (5-CTTTGAACTGTATGGGTCC-3 and 5-CTCCACTGCCTCTGCCTTA-3). As expected, no signal was observed in KO mice.

The *Card15/Nod2^−/−^* (KO) mice used for this study were back-crossed five times with the inbred strains C57BL/6. WT and KO mice were housed and generated in the animal facility at Robert Debré Hospital, Paris, France. The absence of enteropathogens was monitored. All experiments were approved by the institutional committee for animal use.

### Peyer's patches and isolated lymphoid follicle numbers

The entire small intestines were removed and the number of PPs was determinated by macroscopic observation except at birth where PPs were too small to be seen by the naked eye. At birth, small intestines were fixed in formalin, stained with 0.5%methylene blue and decolorized in fresh 2% acetic acid. For LF counts, small intestines were fixed in formalin and rolled up into a ‘Swiss-roll’, embedded in paraffin blocks and cut into 5 µm sections. LFs were counted in a blind fashion on two sections per blocks after haematoxylin-eosin staining.

### Cell composition of Peyer's Patches and spleens

Cell suspensions from PPs were prepared by pressing the three largest PPs for each mouse with a 5 ml syringe piston. The preparation was then incubated with 100 U/ml collagenase D (Roche, Mannheim, Germany) for 30 minutes at 37°C in DMEM media. Cells from spleen were isolated using the same procedure with an additional step of erythrocytes lysis (Gey's-solution). After centrifugation, cells were re-suspended in DMEM media and submitted to flow cytometry analyses on a FACScalibur (Becton-Dickinson), and analyzed by Cell Quest 3.3 (Becton Dickinson). Monoclonal antibodies used to stain cell suspensions were purchased from BD Biosciences (Pharmingen Heidelberg, Germany) : PE-Cy5-anti-CD3 (17A2), PE-Cy7-anti-CD3 (145-2C11), PE-Cy5-anti-CD4 (H129.19), PE-anti-CD4 (RM4), FITC-anti-CD8 (53-6.7), PE-anti-CD11c (HL3), PE-anti-CD25, PE-anti-CD45R/B220 (RA3-6B2), FITC-anti-CD45RB (16A), APC-anti-annexin V and eBioscience (San Diego, CA) : APC-anti-Ly-6G (RB6-8C5).

### Immunohistochemistry

M cells were counted in the FAE using a fluorescence microscope after immunostaining of PP cryostat sections (5 µm) with anti-Ulex Europeaeus antibodies (1/250, 2 h) (Sigma, France), revealed by anti-Ulex Europeaus agglutinin I (1/500, 30 min.) (Vector laboratories) and anti-rabbit FITC conjugate (1/80, 30 min.) (Sigma, France). Caspase 3 immunostaining was done using rabbit polyclonal antibodies to Cleaved Caspase-3 (Asp 175, dilution: 1/100) ( Cell Signaling Technology, Inc Ozyme, Beverly, MA, USA).

### Cytokine Enzyme-Linked Immunosorbent Assay (ELISA)

PPs, ileum and spleen from WT and KO mice were removed, washed with cold PBS and the concentration of protein was determined using commercial kit (Biorad, Marnes la Coquette, France). TNFα, IFNγ, IL-4 and IL-1β were determined by ELISA assays (BD Biosciences) according to the manufacturer's instructions. All experimental groups were tested in duplicates.

### Ussing chamber experiments

Biopsies from ileum with or without PPs were placed in a chamber exposing 0.196 cm^2^ of tissue surface to 1.5 ml of circulating oxygenated Ringer solution at 37°C. PP and ileum permeability were assessed by measuring steady-state (from 1 to 2 h) mucosal-to-serosal flux of 4 kDa FITC-dextran (Sigma, St. Quentin Fallavier, France). Bacterial translocation was studied using chemically killed fluorescein-conjugated *Escherichia coli K-12* or *Staphylococcus Aureus* BioParticles (Molecular Probes, Leiden, the Netherlands) or a viable *Escherichia coli* (the J53 strain resistant to rifampicin) at a final concentration of 1.10^7^ CFU/ml in the mucosal reservoir. *Saccharomyces cerevisiae* translocation was studied using chemically killed fluorescein-conjugated *S. cerevisiae* BioParticles (Molecular Probes, Leiden, the Netherlands) at a final concentration of 1.10^7^ CFU/ml in the mucosal reservoir.

### Real time reverse transcription-polymerase chain reaction (RT-PCR)

After extraction from PP of ileum by the NucleoSpin® RNA II Kit (Macherey-Nagel, Hoerdt, France), total RNA was converted to cDNA using random hexonucleotides and then used for PCR. We conducted PCR with QuantiTect SYBR Green PCR Kit (Applied, Courtaboeuf, France) using sense and antisense primers specific for: G3PDH, 5′-CACCATCTTCCAGGAGCGAG-3′ and 5′-GCCTTCTCCATGGTGGTGAA-3′; Occludin (Occ), 5′-AGCCTTCTGCTTCATCGCTTC-3′ and 5′-GTGGCAATAAACACCATGATGC-3′; Zonula Occludens-1 (ZO-1), 5′- GACTCCAGACAACATCCCGAA-3′ and 5′- AACGCTGGAAATAACCTCGTTC -3′; Zonula Occludens-2 (ZO-2), 5′-CAGCCACAATCAACGTGAATTC-3′ and 5′-CTGTCCTTCAAGCTGCCAAAC-3′. After amplification, we determined the threshold cycle (Ct) to obtain expression values.

### Bacterial content of ileum

The entire ileum (5 cm) was removed and ileal content was collected using 3 mL of steril water (Biorad, France) administered with a polypropylene syringe. Then, ileal content was homogenized and serial dilution (50 µL) of each aliquot were plated onto 5 selective gelose (URI 4, Drigalski, Columbia ANC+5% of sheep blood, Chapman and Coccosel). Plates were incubated for 24 hours at 37°C under aerobic condition and the number of colony forming units was counted and expressed as cfu/mg of ileal content.

### TNBS induced colitis

Under anaesthesia colitis was induced in 12 week old mice by a single intracolonic administration of 120 mg/kg TNBS (Sigma, France) dissolved in 50% ethanol. A 50 µl aliquot of the freshly prepared solution was injected into the colon, 4 cm from the anus, using a 3.5 F polyethylene catheter. The mice were weighed and killed 72 h after TNBS administration. Then, body weight, macroscopic damage score according the Wallace scores [43], and cytokines levels were assessed.

### Statistical Analyses

Values are expressed as mean±SEM. Statistical analysis were performed using GraphPad Prism 4.00 (GraphPad Software, San Diego, CA, USA) software package for PC. Single comparisons were performed by unpaired Student's t-test. A value of P<0.05 was considered as statistically significant. All P values were two sided.

## Supporting Information

Table S1 Impact of Nod2 on body weight, gut weight and intestine length. At weeks 4, 12 and 52, we investigated the body and gut weight and the intestine length of KO and WT mice. No difference was observed between KO and WT mice (P>0.05). Data represent the means±SEM of 8 mice per group.
(0.03 MB TIF)

Table S2 Ileal microflora under basal condition. Under basal condition, no difference was observed between KO and WT mice (P>0.05 for each bacterial group). Data represent the means±SEM of 10 mice per group.
(0.03 MB TIF)

Figure S1 PPs from KO mice exhibit higher rates of CD4+ and CD4-CD8-T-cells at week 4. At week 4, CD3+ T-cells recovered from PPs (A) and spleen (B) were stained with antibodies to CD3, CD4, and CD8 from KO (▪) and WT (□) mice. Data were gated for CD3+ T-cells. Relative proportions of both CD3+CD4+ and CD3+CD4-CD8- T-cells were significantly higher in the PPs but not in the spleen (P>0.05) of KO mice. Data represent the means±SEM of 8 mice per group. *P<0.05; **P<0.01.
(0.08 MB TIF)

Figure S2 Nod2 and CD3+ T -cells in Peyer's Patches. (A and B) Relative proportions of naïve, regulatory and memory T-cells in PPs (A) and spleens (B) of KO (▪) and WT (□) mice at week 12. CD4+ T-cells were stained with antibodies to CD25 and CD45RB. (C) Relative proportions of apoptotic CD3+ and CD3+CD4+ T-cells. Apoptotic CD3+ and CD3+CD4+ T-cells were investigated by flow cytometry using antibodies to CD3, CD4 and annexin V. Data were gated for CD3+CD4+ T-cells. Data represent the means±SEM of 8 mice per group.
(0.04 MB TIF)
