# Supplementary figures and images for: CARD15/NOD2 Is Required for Peyer's Patches Homeostasis in Mice
Source: PLoS One. 2007 Jun 13;2(6):e523. doi: 10.1371/journal.pone.0000523 (PMC1885825; doi:10.1371/journal.pone.0000523)

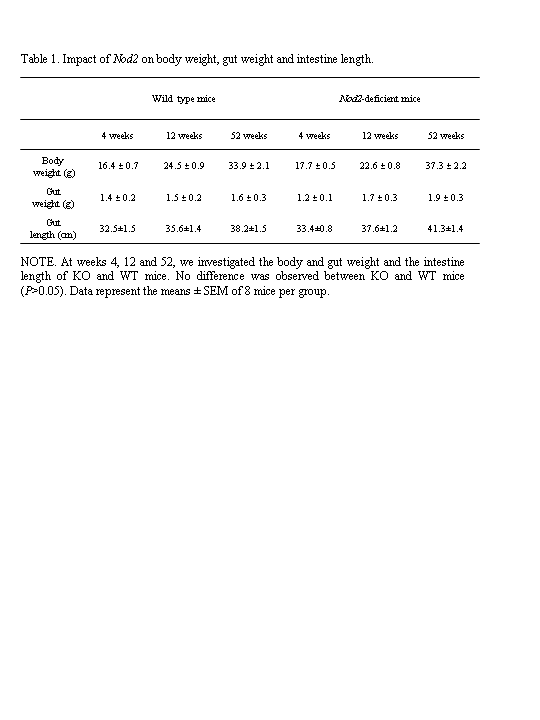

Supplement: Table S1 — Impact of Nod2 on body weight, gut weight and intestine length. At weeks 4, 12 and 52, we investigated the body and gut weight and the intestine length of KO and WT mice. No difference was observed between KO and WT mice (P>0.05). Data represent the means±SEM of 8 mice per group. (0.03 MB TIF) [file pone.0000523.s001.tif]

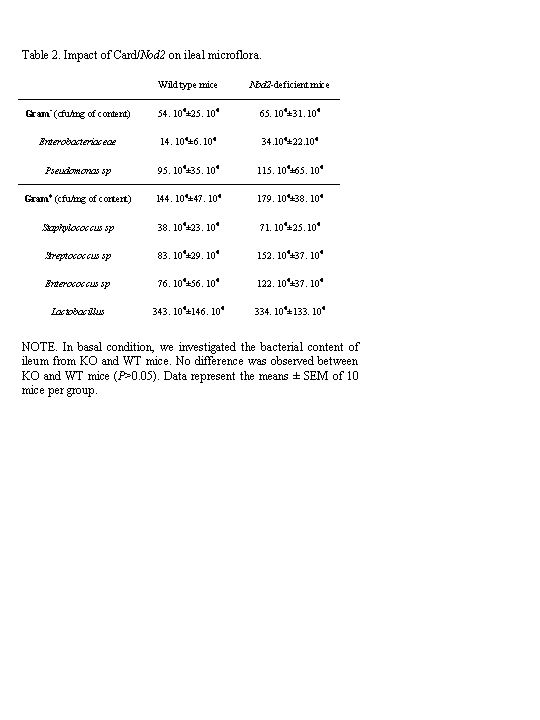

Supplement: Table S2 — Ileal microflora under basal condition. Under basal condition, no difference was observed between KO and WT mice (P>0.05 for each bacterial group). Data represent the means±SEM of 10 mice per group. (0.03 MB TIF) [file pone.0000523.s002.tif]

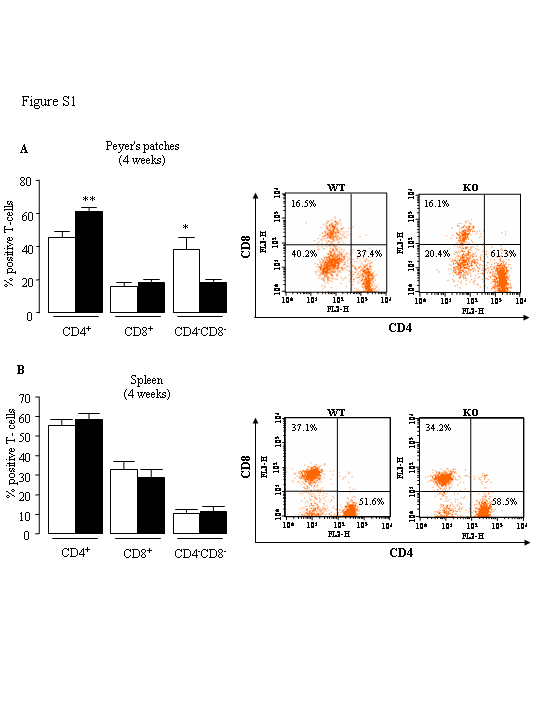

Supplement: Figure S1 — PPs from KO mice exhibit higher rates of CD4+ and CD4-CD8-T-cells at week 4. At week 4, CD3+ T-cells recovered from PPs (A) and spleen (B) were stained with antibodies to CD3, CD4, and CD8 from KO (▪) and WT (□) mice. Data were gated for CD3+ T-cells. Relative proportions of both CD3+CD4+ and CD3+CD4-CD8- T-cells were significantly higher in the PPs but not in the spleen (P>0.05) of KO mice. Data represent the means±SEM of 8 mice per group. *P<0.05; **P<0.01. (0.08 MB TIF) [file pone.0000523.s003.tif]

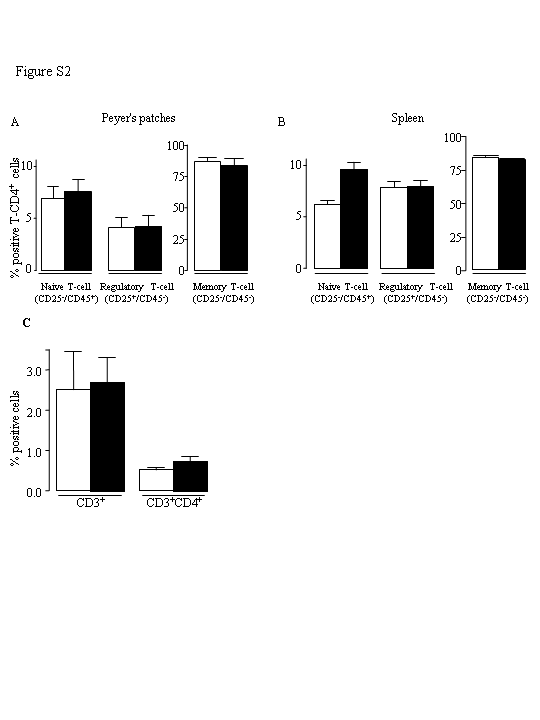

Supplement: Figure S2 — Nod2 and CD3+ T -cells in Peyer's Patches. (A and B) Relative proportions of naïve, regulatory and memory T-cells in PPs (A) and spleens (B) of KO (▪) and WT (□) mice at week 12. CD4+ T-cells were stained with antibodies to CD25 and CD45RB. (C) Relative proportions of apoptotic CD3+ and CD3+CD4+ T-cells. Apoptotic CD3+ and CD3+CD4+ T-cells were investigated by flow cytometry using antibodies to CD3, CD4 and annexin V. Data were gated for CD3+CD4+ T-cells. Data represent the means±SEM of 8 mice per group. (0.04 MB TIF) [file pone.0000523.s004.tif]
